# Risky sexual behavior among people living with HIV/AIDS in Andabet district, Ethiopia: Using a model of unsafe sexual behavior

**DOI:** 10.3389/fpubh.2022.1039755

**Published:** 2022-12-12

**Authors:** Jejaw Berihun Worede, Alemayehu Gonie Mekonnen, Seblewongiel Aynalem, Nakachew Sewnet Amare

**Affiliations:** ^1^Department of Social Work, Bahir Dar University, Bahir Dar, Amara Regional State, Ethiopia; ^2^School of Nursing and Midwifery, Asrat Woldeyes Health Science Campus, Debre Berhan University, Debre Berhan, Amara Regional State, Ethiopia

**Keywords:** sexual practice, HIV/AIDS, unsafe sex, risky behavior, Ethiopia

## Abstract

**Introduction:**

Human immunodeficiency virus (HIV) infection continues to be a major public health problem in Ethiopia. Previous studies have described risky sexual behavior and associated factors among HIV–positive people. These studies, however, did not use a model of unsafe sexual behavior that could address both subjective and objective factors of sexual activity, and there is no study that examines the distal aspects of risky sexual behavior among people living with HIV/AIDS in Ethiopia. Therefore, this study aimed to examine the risky sexual behavior among people living with HIV/AIDS using a model of unsafe sexual behavior.

**Methods:**

An institutional-based study was conducted from March to April 2022. The sample size was determined by using Sloven's formula. In this study, both quantitative and qualitative methods were employed. Study participants were selected using systematic sampling method. An interviewer-administered questionnaire was used to collect the data. Descriptive statistics and correlation tests were computed to analyze the data. The qualitative data was analyzed thematically.

**Results:**

This study included a total of 181 PLWHA clients. The average score for participants' perception regarding the facts of HIV/AIDS was 48.7% (95% CI: 38.9, 58.4). Three months prior to the study, 46.3% of study participants had engaged in at least one risky sexual activity (95% CI: 33.8, 65.4). The correlation model revealed a positive correlation between living in a rural area and risky sexual behavior (*p-*value = 0.001). Furthermore, a poor perception of HIV risks was associated with risky sexual behavior (*p-*value = 0.003). Economic issues, stigma and discrimination, and usage of substances were also identified as contributing factors to unsafe sexual activity in the qualitative data.

**Conclusions:**

A high proportion of PLWHA clients had engaged in at least one risky sexual activity in the 3 months prior to the study. It is not enough to be on ART; additional educational interventions that shape the sexual behavior of PLWHA clients must be considered.

## Introduction

Human immunodeficiency virus (HIV) infection continued to be a major public health problem in Ethiopia (1, 2). The country was one of the fourth countries that reported the highest number of new HIV infected individuals in Africa (3), whereby 22,300 people were newly infected and 690,000 were living with HIV/ acquired immune deficiency syndrome (AIDS) at the end of 2018 (4). HIV/AIDS was responsible for an estimated 34% of all young adult death rates in Ethiopia, and 66.3% of all young adult deaths in urban Ethiopia between the ages of 15–49 (5, 6). The national prevalence of HIV infection in Ethiopia is 0.9% in 2016 (7).

Because of the expansion of antiretroviral therapy (ART) services, people living with HIV/AIDS (PLWHA) are now in better health. A large number of HIV-positive people take ART drugs, and the majority of them believe they are not at risk of transmitting the virus, leading them to engage in risky sexual activity (8). In fact, ART decreases patients' viral loads to undetectable levels, leading to the false impression that they are no longer infectious (9, 10). However, it is important to remember that risky sexual behavior puts people at risk for a range of sexual-related problems such as contracting STIs, unintended pregnancies, and septic abortions (3, 4, 8). Available studies reported that risky sexual practices expose PLWHA clients to new HIV strains resistant to antiretroviral therapy (ART) (5, 11). Accordingly, regardless of whether or not ART is used, adopting safer sex practices is critical for preventing HIV transmission in areas where a substantial number of individuals are living with HIV/AIDS such as Ethiopia (5, 12).

The previous studies have described risky sexual behavior and associated factors among people living with HIV/AIDS. These studies only describe having multiple partners, using condoms inconsistently, sharing needles and syringes to inject drugs, and disclosing status from partners as risky sexual behavior for PLWHA (7, 13, 14). Besides, these studies, however, did not use a model of unsafe sexual behavior that could address both subjective and objective factors of sexual activity.

To the best of our knowledge, there is no study that examines the distal aspects of risky sexual behavior such as societal norms and beliefs about HIV/AIDS, as well as the commoditization of sex as a source of money among people living with HIV/AIDS in Ethiopia. Therefore, this study aimed to examine the risky sexual behavior among people living with HIV/AIDS using a model of unsafe sexual behavior. This model could help better understand risky sexual practices precisely because the model incorporates three interactive components: personal factors, and proximal and distal contexts that best explain risky sexual practices, particularly in the context of HIV/AIDS.

## Methods

### Study design, setting and period

A mixed-method study that included both quantitative and qualitative data was conducted from March to April 2022 in 4 health facilities in Andabet district, Amhara regional state, Ethiopia. The district is located 710 kilometers away from Addis Ababa (capital city of Ethiopia) and 150 kilometers far from Bahir Dar city (the city of Amhara regional state). The district has an estimated total population of 145, 208. Of these, only 3.4% of the population lived in the urban area. Currently, a total of 296 clients registered and started ART in the four health facilities in the district.

### Study population

The study participants were PLWHA who attended their ART in four health facilities; namely Andabet primary hospital, Andabet Health center and Jaragedu health center. The study comprised registered PLWHA clients. Those clients under the age of 18 and those who were seriously ill were excluded from the study.

### Sample size and sampling techniques

The sample size for quantitative data was determined by using Sloven's formula because of its simplicity and the formula assumes a small population variance (15). The formula is given by $\left(n=\frac{N}{1+N{\left(e\right)}^{2}}\right)$ where n is the sample size, *N* is the eligible population size (*N* = 296) and e is the margin of error (0.05). Adding a non-response rate of 10%, the total sample size of 188 participants was selected. Both quantitative and qualitative methods were employed. Study participants were selected using systematic sampling method. Additionally, twelve in-depth interviews (seven female and five male participants) were arranged based on the need of the study.

## Measurements

Risky sexual behavior was assessed by using a model of unsafe sexual behavior. This model is the most commonly used model to explain risky sexual behaviors in the context of HIV/AIDS. It identifies three factors that influence sexual behavior: personal, proximal and distal context (16).

The personal factors (factors that affect risky sexual behaviors from within the person) include the participants' knowledge and perception of HIV risks. Knowledge questions in relation to facts about HIV/AIDS were: What are the ways of HIV transmission? What are the preventive methods for HIV infection? Respondents answered either “Yes” or “No” from the listed options. Based on these questions, participants' knowledge of HIV/AIDS were calculated to classify the respondents into two groups. Respondents who scored the mean and above the mean score of correctly answered questions were classified as knowledgeable, and less than the mean score of correct answers were classified as not knowledgeable about facts in relation to HIV/AIDS.

The participants' perception of HIV/AIDS was assessed by 13 perception-related questions. The questions were three-point Likert-scale items (1 = Agree, 2 = Neutral, and 3 = Disagree). Respondents could answer either “agree”, “neutral” or “disagree” from the listed options. Respondents who scored the mean and above the mean score of the correctly answered questions were classified as having a good perception of HIV risks.

Proximal factors (interpersonal relationships and social conditions) include access to HIV/AIDS information, access to free condoms, getting into forced sexual intercourse, having multiple sexual partners, disclosure of HIV status to a sexual partner(s), talking or introducing condoms during a sexual encounter, ever had sexual intercourse without a condom, and had sex after using substances (drinking alcohol and chewing chat). These variables were assessed by asking study participants about their experience of sexual activity in the 3 months prior to the study. Respondents could answer either “Yes” or “No” and “Yes” answers were considered as having risky sexual activity.

Distal factors include both cultural and structural factors. Cultural factors encompass societal norms and beliefs of the society toward sexual behavior. Structural factors comprise residency (comparison of urban and rural on sexual behavior), and economic status (such as poverty as commoditization of sex). Since these factors are relatively influenced by norms and beliefs of the local community, the variables were explored through an in-depth interview (qualitatively). The interview guide was: Why do PLWHA clients engage in risky sexual activity such as unprotected sex, and commercial sex?

To measure the overall risky sexual behavior of the respondents, the following two variables were used: having multiple sexual partners and inconsistent condom use within 3 months prior to the study. Both variables were measured by “Yes” and “No” questions, and if the respondent's answer was “Yes” for either of the above variables, then he or she was considered as having risky sexual behavior.

### Data collection

An interviewer-administered questionnaire was used to collect the data. The questionnaire was pre-tested and designed first in English and then translated into Amharic (native language). The questionnaires were adapted from previous literature (17–19). Exit interviews were applied by trained enumerators. Five diploma nurses have participated in the data collection. Data completeness was checked by the investigators. For qualitative data, an open-ended guide was prepared to explore perceptions of sexual behavior.

The in-depth interviews were conducted in the local language until no new findings emerged. Study participants were given a subsequent code number based on their registration (respondent number). In addition to audio recording, written notes were taken from the depth interviews.

### Data processing and analysis

The quantitative data were entered into SPSS version 21. Descriptive statistics were computed to explore the data. A correlation test was performed to analyze the data. And finally, the results of the quantitative data were presented in texts and tables with a *p-*value.

For qualitative data, the recorded data were transcribed verbatim in the Amharic language (national language). The typed narratives were then translated into English and verified for accuracy. No computer software was used for qualitative data analysis. The investigators read and reread the transcripts to be familiar with the data and a set of codes were developed to describe groups of categories with similar meanings. The grouped categories were refined and themes were generated from the text data manually. Direct quotations of participants' interviews were presented in italics to highlight key findings.

### Ethical considerations

Ethical approval was obtained from the research and ethical review committee of Bahir Dar University. Written informed consent was obtained from each study participant. All the information obtained from participants was kept confidential throughout the process of study, and the name of the participant was replaced by a code. Withdrawal from the study at any point if they wished was assured.

## Results

### Socio-demographic characteristics of respondents

One hundred eighty-one PLWHA clients were interviewed, with a 96.3% response rate. The mean age of the respondents was 32.4(±3.8 SD) years. Females made up the majority of the respondents (66.3%), while 53.8% of the participants were married. The highest number of study participants (82.8%) were Orthodox Christians, and 63.6% of respondents had no formal education. Nearly half of the respondents (47.4%) were farmers, while more than half (53.6%) were urban residents (Table 1).

**Table 1:** Sociodemographic characteristics of respondents in Andabet district, Ethiopia, 2022.

**Socio-demographic characteristics**	**Categories**	**Frequency (%)**
Age of respondents	≤ 25	39 (21.3)
	26–35	76 (42.2)
	36–45	56 (31.0)
	≥46	10 (5.5)
Sex	Male	61 (33.7)
	Female	120 (66.3)
Marital status	Married	97 (53.8)
	Unmarried	54 (29.8)
	Divorced/widowed	30 (16.4)
Religion	Orthodox	150 (82.8)
	Muslim	23 (12.6)
	Protestant	8 (4.6)
Educational status	No education	115 (63.6)
	Primary	51 (28.2)
	Secondary	8 (4.6)
	College and above	7 (3.6)
Occupational status	Employed	15 (8.3)
	Unemployed	48 (26.5)
	Farmer	86 (47.4)
	Others (Daily laborer)	32 (17.6)
Place of residency	Urban	97 (53.6)
	Rural	84 (46.4)
Lifetime sexual partner	One partner	142 (78.5)
	More than one partner	39 (21.5)

### Personal factors of risky sexual behavior

#### Participants' knowledge of HIV/AIDS

One hundred fifteen (63.6%) of study participants were aware of HIV transmission routes, while 79.4% were aware of HIV infection prevention measures. The most commonly reported HIV prevention method was consistent condom use during sexual intercourse (84.6%). On average, 71.3% of respondents were knowledgeable about HIV transmission and preventive measures (Table 2).

**Table 2:** The respondents' knowledge of HIV/AIDS in Andabet district, Ethiopia, 2022.

**Variables**	**Categories**	
What are the ways of HIV transmission?	Yes, *N* (%)	No, *N* (%)
Through unsafe sexual intercourse	159 (87.7)	22 (12.3)
Sharing needles and syringes	148 (82.1)	33 (17.9)
Blood transfusion	133 (73.4)	48 (26.6)
During pregnancy and childbirth	125 (69.2)	56 (30.8)
Through mosquito and another insect bite	84 (46.3)	97 (53.7)
Casual contact with a person (handshaking…)	41 (22.7)	140 (77.3)
**Average knowledge of HIV transmissions**	**115** (**63.6)**	**66** (**35.6)**
What are the preventive methods for HIV infection?	Yes, N (%)	No, N (%)
Abstain from sexual intercourse	152 (83.9)	29 (16.1)
Consistent use of a condom during sexual intercourse	153 (84.6)	28 (15.4)
Remain faithful to a partner	133 (73.5)	48 (26.5)
Avoid contaminated sharp objects	143 (78.9)	38 (21.1)
Avoid sex with sex workers	138 (76.2)	43 (23.8)
**Average knowledge of preventive methods for HIV**	**144** (**79.4%)**	**37** (**20.6)**
**Overall knowledge of HIV risks**	**Knowledgeable**	**Not knowledgeable**
	129 (71.3)	52 (28.7%)

#### Participants' perception of HIV/AIDS

The respondents' perception of HIV/AIDS was computed by adding up the score of correct responses from thirteen statements. Accordingly, the average score for participants' perception regarding the facts of HIV/AIDS was 48.7% (95%CI 38.9, 58.4) (Table 3).

**Table 3:** The respondents' perception of HIV/AIDS in Andabet district, Ethiopia, 2022.

**Variables**	**Agree**	**Neutral**	**Disagree**
Do you think a person can get HIV the first time he/she has sex?	47 (26.0)	12 (6.6)	122 (67.4)
My HIV status is personal and I will not disclose it to my sexual partner.	48 (26.3)	13 (7.4)	121 (66.3)
I have multiple sex partners because I am already positive and have nothing to fear.	37 (20.4)	15 (8.3)	129 (71.3)
Do you think premarital sex for youths is not supported?	148 (81.8)	10 (5.5)	23 (12.7)
Do you think that disclosing HIV status to a sexual partner affects the relationships?	65 (35.9)	12 (6.6)	104 (57.5)
Do you think that using condoms prevents other types of HIV infection if both sexual partners are HIV-positive?	33 (18.2)	29 (16.1)	119 (65.7)
Do you think that using condoms correctly and consistently reduces the risk of HIV transmission?	129 (71.3)	8 (4.7)	43 (24.0)
Do you think that using a condom is a sign of not trusting a sexual partner?	67 (36.8)	12 (7.0)	102 (56.1)
My sex partner dislikes using a condom during sexual intercourse.	88 (48.5)	42 (23.4)	51 (28.1)
Do you think that someone who has multiple sex partners has a high risk of contracting HIV?	162 (89.5)	2 (1.2)	17 (9.4)
Do you think that an HIV-positive person who begins ART will be cured of the virus?	48 (26.3)	4 (2.3)	129 (71.3)
Do you think that an HIV-infected person can live longer if she or he took ART?	174 (96.1)	3 (1.6)	4 (2.3)
Do you think that after starting ART, an HIV-positive person's need for sexual intercourse will increase?	102 (56.1)	36 (19.9)	43 (24.0)
**Mean score of perception toward HIV/AIDS**	**81** (**48.7)**	**15** (**8.2)**	**85** (**43.1)**

#### Proximal aspects of risky sexual behavior

The proximal aspects of risky sexual behavior were reported by our study participants. As a result, 86.2% of those who participated in the survey had access to HIV/AIDS information. The majority of respondents (69.6%) were forced into sexual intercourse and 20.4% had multiple sexual partners. Nearly three-quarters (73.5%) of study participants talked about condoms during sexual encounters, but 19.3% had sex without a condom. On average, 46.3% of study participants had engaged in at least one risky sexual activity in the 3 months prior to the study (95% CI 33.8, 65.4) (Table 4).

**Table 4:** Risky sexual practices of respondents in Andabet district, Ethiopia, 2022.

**Variables**	**Yes, *N* (%)**	**No, *N* (%)**
Do you have access to HIV/AIDS information in your community?	156 (86.2)	25 (13.8)
Have you ever been forced into sexual intercourse?	126 (69.6)	55 (30.4)
Do you have multiple sexual partners?	37 (20.4)	144 (79.6)
Have you ever disclosed your HIV status to your sexual partner before having sex?	133 (73.5)	48 (26.5)
Do you have access to free condoms in your community?	32 (17.7)	149 (82.3)
Do you talk about or introduce condoms during sexual encounters?	133 (73.5)	48 (26.5)
Have you ever had sexual intercourse without a condom?	37 (19.3)	146 (80.7)
Have you ever did sex while you had drunk alcohol?	49 (27.1)	132 (72.9)
Have you ever did sex while you had chewed chat?	44 (24.3)	137 (75.7)
**Average risky sexual practices**	**84** (**46.3)**	**97** (**53.7)**

#### Distal aspects of risky sexual behavior

Distal aspects of risky sexual behavior include societal norms and beliefs about HIV/AIDS, as well as the commoditization of sex as a source of money. We used in-depth interviews to explore the data because these factors are influenced by the norms and beliefs of the local community. Study participants were interviewed individually. The interview questions were: Why do PLWHA clients engage in risky sexual activity such as unprotected sex, and commercial sex? Three themes have emerged from the data: Economic issues, the presence of stigma and discrimination and the use of substances. All of the reported findings are taken inductively from participants' responses and described below with quotations for each theme.

##### Theme one: Economic issues

The majority of young female PLWHA clients in the study area did not have a reliable source of income to lead their life properly, and they intentionally engaged in commercial sex to get money. For example, an interviewee (R6) said that:

“*Sometimes, I have had sexual intercourse to get money. Because I did not have any other way to support myself, and I could not say no to sex if a man was willing to pay for it. For example, I know that my close girlfriend also has sex with many men in order to gain money, she does not have any other source of income other than this… ”*

Another interviewee (R4) also said that:

“*I have a child whose father no longer lives with me. I have sexual relationships with other men, just to get income. To make money, I have been doing commercial sex to …”*.

##### Theme two: The presence of stigma and discrimination

The study participants also highlighted how stigma and discrimination contribute to unsafe sexual behavior. When there is high stigma and discrimination in the community about being HIV-positive, PLWHA clients tend to exclude themselves from the community and even fear taking free condoms from health facilities. In this regard, an interviewee (R7) reported that;

“*I wanted to get a condom from the health clinic, but I was afraid that others would know I was HIV positive, so I did not get a condom and had intercourse without it…He added… I understand that unprotected intercourse is a dangerous sexual behavior that might lead to HIV transmission”*.

##### Theme three: Use of substances (drinking alcohol, chewing chat)

The power of substances to stimulate the person to undertake unsafe sex was well explained by our study participants. One participant (R3) stated that:

“*Drinking alcohol motivates people to have sexual intercourse with HIV-positive people or healthy people. He could then become infected or spread HIV to others... Chewing chat has the same effect... stimulate to engage in unsafe sex”*.

Our interviewees also pointed out how using substances such as drinking alcohol and chewing chat could push risky sexual practices. An interviewee (R9) said that:

“*In our area, chat chewing is not as common as drinking alcohol. HIV-positive people have engaged in unsafe sexual intercourse with many people after they drink alcohol. This could then spread HIV to others*...

#### Factors associated with risky sexual behavior

A correlation test was performed between risky sexual behavior and sociodemographic variables. The correlation model revealed a positive correlation between living in a rural area and risky sexual behavior (*p-*value = 0.001). Furthermore, a poor perception of HIV risks was associated with risky sexual behavior (*p-*value = 0.003) (Table 5).

**Table 5:** Correlation test between risky sexual behavior and sociodemographic variables, 2022.

**Variables**		**Risky sexual behavior**		**Pearson correlation coefficient**	***P*** **-value**
		**Yes**	**No**		
Sex of respondents	Male	37	24	0.209	0.070
	Female	85	35	1	
Place of residency	Urban	65	32	0.701	0.001
	Rural	57	27	1	
Perception of HIV risks	Poor	69	36	0.630	0.033
	Good	53	23	1	

## Discussion

Personality factors play an important role in determining a person's sexual behavior (11, 20). Previous studies, for example, have revealed that people's knowledge and perceptions of HIV/AIDS facts influence risky sexual behavior among PLWHA clients. In this study, on average, 71.3% of respondents were knowledgeable about HIV transmission and preventive methods, whereas participants' perceptions of HIV risks were 48.7%. Previous studies have reported that PLWHA clients have a poor perception of HIV risks, which is consistent with our findings (21, 22). This finding adds to the growing body of evidence that HIV- positive clients who start ART tend to have a poor perception of HIV risks.

Even though 78.5% of respondents had talked about condoms with their partners, only 19.3% of them had sex without a condom. This suggests that there are significant gaps in the practice of safe sexual conduct among PLWHA that needs practical intervention. On average, 46.3% of study participants had engaged in at least one risky sexual activity within the 3 months prior to the study. Relatively, similar figures were reported from Addis Ababa (39.1%) (8), western Oromia (56.9%) (4) and Kembata Tembaro zone (40.9%) (23). It is, however, lower than reports from Gambella town (79.8%) (19). This discrepancy could be attributable to differences in study settings and measurements and definitions of risky sexual behavior among studies. Regardless of these variations, behavioral interventions that can reduce unsafe sexual practices among PLWHA should be emphasized.

In this study, societal norms and beliefs about HIV/AIDS, as well as the commoditization of sex as a source of money were explored through in-depth interviews as data triangulation is the best method to generate valid and more comprehensive findings. Economic concerns, stigma and discrimination, and substance abuse were the three themes that emerged from the study. Accordingly, our study participants lacked a stable source of income and engaged in commercial sex, which is a known risk factor for contracting a new strain of HIV (21).

Our study participants also mentioned that stigma and discrimination as contributing factors to unsafe sexual activity. In agreement with our study, previous studies have reported that perceived stigma and discrimination influence HIV/AIDS clients' ability to receive effective treatment (24). If there is a high stigma and discrimination toward HIV-positive status in the community, PLWHA clients tend to exclude themselves from the community and even fear taking free condoms from health facilities (25, 26). In addition to economic issues and the presence of stigma and discrimination, use of substance use of substances has the power to stimulate the person to undertake actions like unsafe sex (27). In this regard, our interviewees' pointed out how using substances such as drinking alcohol and chewing chat could push risky sexual practices. There is also evidence that heavy alcohol consumption can be linked to unprotected sex (14, 28). This means that being on ART is not enough; extra educational interventions that shape PLWHA clients' sexual behavior must be considered.

In this study, a correlation test was performed to see the association between risky sexual behavior and sociodemographic variables. The correlation model revealed a positive correlation between living in a rural area and risky sexual behavior. Moreover, a poor perception of HIV risks was associated with unsafe sexual conduct. A similar report was obtained in research conducted in Ghana (29), Nigeria (30) and Southern Ethiopia (21).

## Limitations of the study

This study has some limitations that must be acknowledged. Quantitative results from a small sample size may be less reliable. Since there is no standardized definition of risky sexual behavior, the results of this study should be interpreted cautiously. The authors also have limitations associated with individuals' socially desired responses rather than true feelings.

## Conclusions

High proportions of PLWHA clients had engaged in at least one risky sexual activity in the three months prior to the study. It is not enough to be on ART; additional educational interventions that shape the sexual behavior of PLWHA clients must be considered.

## Data availability statement

The original contributions presented in the study are included in the article/supplementary material, further inquiries can be directed to the corresponding author.

## Ethics statement

The studies involving human participants were reviewed and approved by the Research and Ethical Review Committee of Bahir Dar University. The patients/participants provided their written informed consent to participate in this study.

## Author contributions

JW, AM, SA, and NA participated in the design of the study, supervised the data collection, analyzed, and interpreted the data. AM drafted and edited the manuscript. All authors reviewed and approved the final manuscript.
